# A Nominalist Alternative to Reference by Abstraction

**DOI:** 10.1111/theo.12399

**Published:** 2022-02-28

**Authors:** Gareth Rhys Pearce

**Affiliations:** ^1^ Faculty for Philosophy University of Vienna Universitätsstraße 7 Vienna 1010 Austria

**Keywords:** nominalism, reference by abstraction, philosophy of mathematics, thin objects

## Abstract

In his recent book Thin Objects, Øystein Linnebo (2018) argues for the existence of a hierarchy of abstract objects, sufficient to model ZFC, via a novel and highly interesting argument that relies on a process called dynamic abstraction. This paper presents a way for a nominalist, someone opposed to the existence of abstract objects, to avoid Linnebo's conclusion by rejecting his claim that certain abstraction principles are sufficient for reference (RBA). Section 1 of the paper explains Linnebo's argument for RBA. It offers a reading of Linnebo's work upon which he has two arguments for RBA: one deductive and one abductive, and argues that whilst the deductive argument is unsound the abductive one is prima facie plausible. The nominalist must therefore find a way to respond to the abductive argument. Section 2 outlines just such a response, by offering an alternative explanation of the cases Linnebo wishes to argue from. Most interestingly, it shows that abstraction in Linnebo's most difficult case (the “reference to ordinary bodies” case) can be achieved using mereological means, rather than relying on RBA.

## 1 INTRODUCTION

In his recent book *Thin Objects*, Øystein Linnebo (2018) argues for the existence of a hierarchy of abstract objects via a novel and highly interesting argument that relies on a process called dynamic abstraction.^1^ A crucial premise in Linnebo's argument for dynamic abstraction is his claim that predicative abstraction principles succeed in securing reference. This premise is as follows:

**Reference by Abstraction (RBA):** Let ∼ be any (partial) equivalence relation over an established domain of first‐ or higher‐order entities *D*. Define a function *f* with *dom*(*f*) ⊆ *D* such that ∀*x*, *y* ∈ *D* (*f*(*x*) = *f*(*y*) ↔ *x* ∼ *y*). *ran*(*f*) need not be a subset of *D*. It is always the case that *f*(*x*) genuinely refers to some entity. Typically, this entity is not in *D*.

As Linnebo shows in *Thin Objects*, this premise, along with a number of other plausible premises, entails the existence of abstracta (Section 2.1).

Nominalism is a family of views characterised by opposition, in some respect, to the existence of abstract objects.^2^ For instance, three types of nominalist are: ontic nominalists who believe that there are no abstract objects, epistemic nominalists who believe that one cannot be justified in believing that there are abstract objects, and semantic nominalists who think talk of abstract objects is in some sense faulty. This distinction follows Burgess and Rosen's well‐known work (1997) on nominalism in the philosophy of mathematics. Clearly, nominalism of any form is incompatible with Linnebo's conclusion that there are abstract objects.^3^

This paper explains how an ontic nominalist (henceforth just *nominalist*, unless specified otherwise) may avoid Linnebo's argument by rejecting **RBA**. Section 2 briefly explains **RBA** and its role in Linnebo's argument. It continues by drawing on textual evidence in both *Thin Objects* and Linnebo's Aristotelian Society paper *“Reference by Abstraction”* Linnebo (2012) to offer a reading of Linnebo's work on which he offers two arguments for **RBA**: one deductive and one abductive. Section 2 argues that the deductive argument is unsound but that the abductive argument is plausible. The challenge to the nominalist, therefore, is to respond to the abductive argument. Section 3 attempts to meet this challenge by giving alternative explanations of the kinds of cases Linnebo presents.

Exactly what the presence of a viable nominalist explanation of the facts at hand means for Linnebo's view depends on one's views about abduction. The classical view of abduction is inference to *the* best explanation (Lipton, 1991). In this view, an abductive inference to an explanation is valid iff the inference is the singular best explanation of the facts to be explained. In this view of abduction, if the nominalist explanation is as good as Linnebo's, then it would deny him his conclusion. But there is no reason to assume such a conservative view of abduction. It might be the case, for instance, that any abductive inference to a reasonable and non‐dominated explanation of the facts is valid. In this view, if Linnebo's explanation and the nominalist's are comparably good; this might be a case of rational disagreement. The parties can amicably disagree, and both go on believing what they did before. Either way, if the nominalist can show that they can explain Linnebo's cases as well as he can, they are able to avoid his conclusion. This paper leaves open what this means for the validity of Linnebo's inference.^4^

The paper therefore proceeds as follows. Section 2 explains **RBA**, its role in Linnebo's wider argument, and Linnebo's arguments for it (one deductive, one abductive). Section 3 presents a rival nominalist explanation of the cases that support **RBA**, thus giving the nominalist a way out of Linnebo's argument for abstract objects.

## 2 REFERENCE BY ABSTRACTION

### 2.1 RBA and dynamic abstraction

Dynamic abstraction is the process by which Linnebo argues for and purportedly secures the existence of new abstract objects. The argument is as follows:

P1 If it is possible to refer to some *o*, then *o* exists.
P2 Let ∼ be any (partial) equivalence relation over an established domain of first‐ or higher‐order entities *D*. Define a function *f* with *dom*(*f*) ⊆ *D* such that ∀*x*, *y* ∈ *D* (*f*(*x*) = *f*(*y*) ↔ *x* ∼ *y*). *ran*(*f*) need not be a subset of *D*. It is always the case that *f*(*x*) genuinely refers to some entity. Typically, this entity is not in *D*.^5^(**RBA**)
P3 It is possible to re‐apply many of the abstraction principles of the above form to the new objects to generate further objects, repeating the process. One such principle, when iterated, generates a model of Zermelo‐Fraenkel set theory with Choice (ZFC).
C Abstract objects exist, some of which constitute a model of ZFC.

As an example, consider the following abstraction principle which relates lines (which are taken to be concrete spacetime regions) and directions: for any two lines *l*_1_ and *l*_2_, *dir*(*l*_1_) = *dir*(*l*_2_) ↔ *l*_1_||*l*_2_, where *dir* is the function that defines directions and || is the relation of parallelism between lines.

The function *dir* satisfies the conditions for **RBA**. It is a partial equivalence relation over an established domain of entities (namely lines). In this case, the range of *dir* is not in *D*; directions are yet to be established. By **RBA**, it follows that for any line *l*, “*dir*(*l*)” genuinely refers to some object. Because *ran*(*dir*) is disjoint from *D*, “*dir*(*l*)” refers to something not in *D*. But by P1 it follows that this object must exist as it is the referent of “*dir*(*l*).”

In this case, iteration is trivial (directions are parallel only to themselves), but in other cases, such as abstraction principles for sets, the principles can be re‐applied to the new domain of objects, generating even more objects.

Clearly, **RBA** is a central premise in this argument, and the one to which this paper objects.

### 2.2 Linnebo's argument for RBA

Sadly, Linnebo never explicitly signposts what his argument for **RBA** is. That is not to say he does not have an argument for it, he certainly does, but it is a non‐trivial exegetical question exactly what its structure is. This section offers two possible arguments that can be pulled from Linnebo's work: one deductive and one abductive. The section entitled “The deductive argument” also argues for the substantive claim that the deductive argument, if it is what Linnebo has in mind, is unsound. This interpretation draws both on *Thin Objects* (Linnebo, 2018) and *“Reference by Abstraction”* (Linnebo, 2012).

#### The deductive argument

There are some sections of *Thin Objects* where Linnebo might be read as claiming that his so‐called flexible conception of ontology entails **RBA** (ibid. pp. 31,32). This section explains what this argument would be and sets out the textual evidence that it might be an argument Linnebo has in mind. It then argues that the argument is unsound.

The rigid conception of ontology can be described as a “Lego blocks” conception of ontology. On this view *“reality is ’carved up' into objects in a unique way that is independent of the concepts that we bring to bear”* (p. 31). On the rigid conception, reference is required to adhere to this objective structure in some manner.

On the other hand (p. 31): “The flexible conception … insists that reality is articulated into objects only through the concepts that we bring to bear. And we often have some choice in this matter.” Reference, and hence existence, becomes a much lighter notion on this view.

The exact content of the rigid and flexible conceptions is left a little vague by Linnebo. He says little more than what is reported here and never makes the claims of these views entirely clear and specific. This is not necessarily a problem. Perhaps what Linnebo's doing is setting out two broad ways of approaching ontology rather than exact doctrines. The idea behind the two views is clear enough. For present purposes, this paper understands the flexible conception as stating that if there is a way of specifying a portion of reality, then that is sufficient for that portion being an object. There are no stronger demands on something being an object than being a specifiable portion of reality. The rigid conception states that not all specifiable portions of reality are objects, and that there are certain sufficiently conservative additional constraints that must also be met. This is, again, not a complete description of the views. Exactly what portions of reality are, what specification is, or what the sufficiently conservative additional constraints on the rigid conception are, is left unexplored. But this is enough of a working description of the view for present purposes.

There is some textual evidence that Linnebo thinks that the flexible conception entails **RBA**. In section 6.5 of *Thin Objects*, entitled “Internalism about Reference,” Linnebo presents a possible nominalist response to **RBA**. In this, he says the following:

The comparison with the case of physical bodies brings out some important lessons. First, the nominalist's challenge is just an instance of a far more general skeptical challenge concerning what it takes to specify an object. It isn't the abstractness of the desired object that is fueling the challenge but some very general preconceptions about what it takes to specify an object. The Fregean response is to reject these preconceptions as unreasonable. (ibid. p. 42)

Linnebo simply refers to this as *the* nominalist response. This is unfortunate because this paper's nominalist response seems to have very little in common with Linnebo's nominalist. This paper does not endorse the way in which Linnebo's nominalist responds to **RBA**. For clarity's sake, this paper refers to the nominalist view outlined in section 6.5 of *Thin Objects* as “Linnebo's nominalist," to provide distance from that view and its own. Any quoted text remains unchanged, however.

One way to read Linnebo's claim is to understand the Linnebo's nominalist's worries as presupposing the rigid conception of ontology. This would certainly be a commitment that would block Linnebo's argument, though potentially a heavy‐handed one. The flexible conception is a necessary condition on **RBA** because simply specifying a prospective object is not enough to refer to said object, given the rigid conception. What is contested is if the flexible conception is sufficient for **RBA**. When Linnebo says *“the nominalist's challenge is just an instance of a far more general skeptical challenge concerning what it takes to specify an object,"* he appears to be claiming that one rejects **RBA** iff one accepts the rigid conception of ontology. Consequently, the flexible conception of ontology entails **RBA**. But, so argues Linnebo, the flexible conception is true as the rigid conception places unreasonable demands on ontology. Formally, Linnebo's argument would be as follows:

P1: If the flexible conception of ontology is true, then the rigid conception of ontology is false.
P2: The rigid conception is true iff **RBA** is false.
P3: But the flexible conception is true
C: So, **RBA** is true

This is a non‐trivial exegetical claim. Linnebo never explicitly endorses an argument of this form. It may very well be the case the Linnebo simply has the abductive argument in mind and the flexible conception of ontology is intended as a necessary, rather than necessary and sufficient, condition on **RBA**.

If this is Linnebo's argument, then it is unsound. The flexible conception of ontology is merely a necessary, not sufficient, condition on **RBA**. **RBA** can be false without invoking the rigid conception of ontology.

Consider the following view that accepts the flexible conception while rejecting **RBA**. Suppose one thinks the world is made up of a concrete (non‐abstract) property mosaic with properties like charge, mass, or spin (properties instantiated by concrete objects) rather than properties or relations like membership or succession (properties instantiated by abstracta). One allows arbitrary reference over the mosaic. Any way of arbitrarily carving up the mosaic generates an object. This means that any specifiable portion of reality is an object. Hence, the flexible conception is true on this view. However, this view would reject **RBA** because **RBA** entails the existence of certain abstracta such as sets, which do not exist on this view. Hence, it is not the case that **RBA** is false iff the rigid conception is true.

That is not to say that this is how nominalists have to look at the world. The argument is just to show that there are ways of accepting the flexible conception of ontology whilst rejecting **RBA**. But if there are ways of accepting the flexible conception whilst rejecting **RBA**, then the flexible conception does not entail **RBA**.

Thus, if this is the argument intended by Linnebo, it is unsound. Consequently, the case for **RBA** rests on the abductive argument.

#### The abductive argument

Linnebo gives a series of puzzling cases that seem to be examples of **RBA**. In *Thin Objects*, Linnebo presents the case of reference to ordinary physical bodies (ch2, section 3, pp. 26–31), the case of reference to book tokens versus types (ch2, section 4, pp. 32–33), and the case of reference to directions (ch2, section 5, p. 34). In *“Reference by Abstraction"* (Linnebo, 2012), Linnebo also presents the case of reference to inscriptions (letter tokens) and letters. This is analogous to the book case in *Thin Objects*. Accepting **RBA** would explain what happens in all of these cases. There are two reasons to think that Linnebo has an abductive argument in mind.

First, Linnebo's Aristotelian Society paper (2012) defends reference by abstraction and is explicitly abductive. In this paper, Linnebo sets out a puzzling case, considers an abstractionist and a nominalist explanation of this case, and then argues that his abstractionist explanation has certain theoretical and explanatory virtues that the nominalist explanation lacks. The case he considers is ordinary talk about individual letter inscriptions (i.e., the literal body of ink left on a page) and letter types (i.e., the type or class of all inscriptions expressing the same letter). For example, this inscription of the name “Anna” contains four inscriptions but two letter types. Prima facie, the last sentence quantifies over (1) the four literal characters in the name and (2) two other objects, the letter types. The puzzle is to explain what is going on in sentences like the one above. Nothing hangs on using inscriptions and letter types specifically; the argument works just as well with any type of thing and its particular token instances.

Second, Linnebo's response to his nominalist (ibid. section 6.5) appears abductive in nature. In his discussion of ordinary bodies, Linnebo sets out a “model” (Linnebo avoids calling this a full account) of reference to these bodies via abstraction (ch2, section 3, pp. 26‐31). On this model, one defines ordinary medium‐sized objects using a kind of bodily contiguity relation on mere parcels of matter. For instance, two parcels of matter are bodily contiguous iff they are connected via solid matter, move as a relatively uniform block when force is applied, are enclosed within the same natural boundaries, and so forth. Let ∼ be this bodily contiguity relation, define *body*(*x*) = *body*(*y*) ↔ *x* ∼ *y*.

Linnebo's nominalist's response is simply to refuse to accept that there are abstract objects (in this case directions) to which one could refer via abstraction. About this response, Linnebo says the following:

Clearly, [the nominalist and I] are confronted with a fundamental disagreement about what it takes to specify a direction. To break the impasse, it is useful to consider a structurally analogous debate that arises in the case of physical bodies. Here too I claim to have provided an account of reference. To specify a physical body, it suffices to have causally interacted with one of its parts and to be operating appropriately with the relevant unity relation. Assume someone challenges me to demonstrate that there really exists a physical body associated with some parcel of matter that both parties admit exists and is in the field of the unity relation. The challenger demands that the alleged referent be shown to her in a more direct or secure way that she too would find acceptable. Clearly, there is nothing I can do that would satisfy the challenger. A physical body just is the sort of thing that is most directly specified by means of a parcel of matter and is subject to the appropriate unity relation. To demand that a body be specified or shown in some altogether different way is to demand the impossible. (ibid. ch 2. pp. 42)

I think we should understand Linnebo's argument as saying that the principle works in the case of physical bodies (and other cases), so this should be taken as evidence that the principle holds more generally. It would be potentially ad hoc to think that it holds in this case but not generally. As such, the best explanation of why the principle holds in the case of physical bodies is that **RBA** holds generally.

A viable response for the nominalist, then, is to explain what goes on in these cases without relying on **RBA**. If the nominalist can provide a comparatively good explanation, they can reasonably reject Linnebo's second premise. This is the strategy Section 3 adopts.

## 3 NOMINALIST EXPLANATIONS OF APPARENT CASES OF ABSTRACTION

Across *Thin Objects* (Linnebo, 2018) and *“Reference by Abstraction”* (Linnebo, 2012), Linnebo gives four examples of cases where reference by abstraction apparently takes place. These cases are ordinary physical bodies (ibid. ch2, section 3, pp. 26‐31), book tokens and types (ibid. ch2, section 4, pp. 32–33) and directions (ibid. ch2, section 5, p. 34), and inscriptions (letter tokens) and letters (Linnebo, 2012).

These cases can be split into two families:

Directions, letters, and book types are all instances of apparent direct reference to abstracta in ordinary talk. These can all be thought of as types of objects. Directions are line‐types and letters are inscription types.

The case of reference to ordinary bodies cannot be handled in this way. Ordinary bodies are not *types* of parcels of matter; they are parcels of matter. Moreover, whereas the nominalist wishes to reject the existence of types (or, at least, reject their existence as abstract first‐order entities), they have no reason or obvious desire to do away with ordinary bodies.

Different nominalist explanations will be offered in each of these cases. Section 3.1 deals with reference to types, and Section 3.2 deals with reference to ordinary bodies.

### 3.1 Reference to types

Talk of types is ubiquitous in everyday language. Two people may read the same book despite reading token‐different books. Two people may write the same word despite making separate inscriptions. Two people might have the same (kinds of) pet despite having token‐different pets. It appears, on the face of it, that reference to things called types happens all the time. The explanation offered by Linnebo as to how this happens is via abstraction. Whenever there is a type, there is a certain sort of similarity relation that constitutes a partial equivalence relation. Not any similarity relation will do as similarity is, generally, not transitive. But some are transitive and hence acceptable, for instance, two books having the same sequence of words. For non‐transitive similarity relations, there are technical tricks that can be used to define a transitive similarity relation over them.^6^

If one accepts **RBA**, then it is clear to see how this leads to reference to an abstract object called a type. If *sim*_*T*_ is the transitive similarity relation governing some family *T* of types, then *T*(*x*) = *T*(*y*) ↔ *sim*_*T*(*x*,*y*)_. By **RBA**, there really is reference to an abstract type.

The nominalist option is either to accept that there are types but provide an alternative explanation as to how reference to them happens, or to reject the existence of types but find a way of explaining type‐talk despite the lack of reference to types. Clearly, the nominalist has to accept the latter option.

Ironically, it is the existence of the very equivalence relation necessary to perform abstraction that allows the nominalist to explain away reference to types. The nominalist strategy is to paraphrase literal type talk as non‐type talk using *sim* in an appropriate way. For instance, “we read the same book” can be paraphrased as “the books we read are appropriately similar with respect to their content and sequence of inscriptions” or “we have the same type of pet” as “the pets we have are similar with respect to the appropriate biological similarity relation.”

But this is not the only type of type‐talk, and different instances might require different ways of paraphrasing. Consider, for instance, the claim “this is a copy of Anna Karenina” or generally that “x is an instance of type T.” The way the nominalist can handle this sort of case is by generating new predicates from the equivalence relation. If *T* is a family of types under the same equivalence relation *sim*_*T*_ and *α* is an archetypal instance of an intended type, then one then defines a unary predicate *P*_⟨*T*,*α*⟩_(*X*) as being short‐hand for *sim*_*T*(*x*,*α*)_. For example, if *AK* is an archetypal copy of Anna Karenina (e.g., the first copy written), then “this is a copy of Anna Karenina” translates to “*P*_⟨*books*,*AK*⟩_ (where “books” is a family of types generated by the appropriate book‐similarity relation). Similarly apparent properties of types can be interpreted by quantifying over the tokens of that type: “Anna Karenina is a work of fiction” can be paraphrased as $\forall x\;\left(P_{\left\langle \text{books},\mathrm{AK}\right\rangle }\left(x\right)\to \mathrm{Fic}\left(x\right)\right)$.

Linnebo anticipates this response in *“Reference by Abstraction”* (Linnebo, 2012) under the name of semantic reductionism (labelling his own view “semantic non‐reductionism”). Rather than arguing that semantic reductionism is wrong, Linnebo argues simply that his non‐reductionist view is preferable. Linnebo gives two reasons for thinking this.

The first reason is that the semantic reductionist cannot find a suitable paraphrase of mathematical claims about types. Consider, for instance, the claim “there are more copies of Harry Potter than Anna Karenina.” A natural way to phrase this is to say something like *P*_⟨*books*,*HP*⟩_ > *P*_⟨*books*,*AK*⟩_, for which *HP* and *AK* are archetypes of the respective books, and the *P* terms are second‐order entities. But there is a very real accusation that this is, to use the Quinean mantra, set theory in disguise.

An important point to note is that the puzzle here is not about reference to types, per se, but how the nominalist is to account for everyday uses of mathematics. This is a well‐known problem for nominalists and was raised by Quine (1953). There are many strategies available that span the spectrum of nominalist positions. For instance, strategies might involve fictionalism (Leng, 2010) or eliminative structuralism (Hellman, 1989). To sketch one such solution, a nominalist might adopt a particular reading of higher‐order logic that does not involve sets (e.g., that of Boolos (1984)) and interpret these kinds of basic everyday mathematical statements in higher‐order logic. For instance, the claim *P*_⟨*books*,*HP*⟩_ > *P*_⟨*books*,*AK*⟩_ can be understood as a claim about the “existence” (in the higher‐order, not first‐order, sense) of an injective function from the copies of Anna Karenina to a subset of the copies of Harry Potter, and no bijection between them.

Suffice to say that which, if any, of these strategies work, is a much wider question about the viability of nominalist approaches to mathematics that this paper could not hope to do justice to. The nominalist has well developed and promising tools for handling these kinds of cases. There is nothing this paper could briefly add to what is an extensively debated open question in the philosophy of mathematics. This paper simply defers to existing solutions on the matter.

Linnebo's second argument is that the semantic reductionist is committed to a strange outcome in the case of ordinary bodies. For instance, if semantic reductionism is to be applied generally by *the nominalist*, then they are committed to saying that really there is no reference to physical bodies, only a paraphrase of bodily contiguity claims about parcels of matter. This, says Linnebo, violates ordinary speakers' understanding of their language.

Linnebo's objection is correct if semantic reductionism is applied in all cases. But there is no reason why the nominalist needs to do that. The nominalist has many tools for avoiding reference to abstract objects, and some of them are applicable in some cases but not in others. The nominalist strategy is disunified, and this is the bullet they must bite in exchange for the virtues of parsimony and avoiding abstracta. What the nominalist can say about ordinary bodies is discussed in the next subsection.

### 3.2 Reference to ordinary bodies

In this case, Linnebo argues that reference to ordinary medium‐sized bodies can be secured via abstraction over parcels of matter. One does this by defining a bodily contiguity relation ∼ such that two parcels of matter are bodily contiguous iff they are connected via solid matter, move as a relatively uniform block when force is applied, are enclosed within the same natural boundaries, and so forth (Linnebo gives a full set of conditions in the book). One then defines the natural abstraction principle *body*(*x*) = *body*(*y*) ↔ *x* ∼ *y* and, according to Linnebo, one then employs **RBA** to guarantee reference to ordinary bodies.

As with types, the nominalist has two options: they can reject that this process actually does lead to reference to ordinary bodies, or they can find another explanation of how this reference happens, aside from via **RBA**. Linnebo's nominalist appears to want to take the first option, but this is a mistake for exactly the reasons Linnebo outlines in *“Reference by Abstraction”* and *Thin Objects*. This just does seem to be a perfectly good case of reference. And, unlike with abstract types, the nominalist has no need to eliminate ordinary objects. Reference by abstraction can be nominalistically acceptable when the resulting object is concrete, as in the ordinary bodies case. The challenge, therefore, is to find an explanation as to why reference by abstraction works in these cases without appealing to **RBA**, the claim that reference by abstraction is generally successful.

#### Reference by mereology

Reference in the case of abstraction over ordinary bodies can be achieved via mereological means. This exploits a crucial difference between the case of ordinary bodies and other cases, such as abstraction to sets or directions. In abstract cases, such as sets or lines, the new objects are equivalence classes of some prior established entities. In the ordinary bodies case, however, the new objects are mereological sums, not classes, of the established entities. As such, it is possible to use mereological tools to recover reference by abstraction in the case of ordinary bodies.

This result can be shown with only two axioms: the axiom of fusion (AoF) and the axiom of antisymmetry.

This paper uses a formulation of the axiom of fusion inspired by Varzi and Cotnoir's definition (Varzi & Cotnoir, 2021, section 3.1.3).^7^ Define a mereological fusion as follows. Let *z* be an object and *ϕ* a formula with at least *x* free and all other free variables being *v_1_,…,v*_*n*_ Let ≼ be the parthood relation. *z* is the mereological fusion of the *ϕ*s iff (∀x(φ(x)→x≼z) ∧ ∀y(∀x(φ(x)→x≼y)→z≼y)). The first conjunct says that *z* contains all the *ϕ*s as parts, and the second says that it is the least entity to do so. We write *F*_*ϕ*_(*z*) to say that *z* is the mereological fusion of the *ϕ*s. Clearly, for any *ϕ* with free variables other than *x*, *F*_*ϕ*_(*z*) can only be true of *z* given a variable assignment. The axiom of fusion is the claim that for all *ϕ* with v_1_,…,v_n_, x free, ∀v_1_,…,∀v_n_(∃x φ→∃z F_φ_(z)). The Varzi‐Cotnoir formulation of the axiom requires *ϕ* to only have *x* free. If we move to languages where every object is the referent of some term, then the two formulations collapse into one another.

Antisymmetry is the claim that ∀x∀y((x ≼y∧y ≼x)→x=y).

Let ∼ be a partial equivalence relation over an established domain of objects. Let *ϕ* be the formulae *x* ∼ *y*. It follows by the axiom of fusion that ∀*x* (∃*y x* ∼ *y* → ∃*z F*_*x*∼*y*_(*z*)). It follows from antisymmetry that mereological fusions are unique. Let for any *ϕ* let *F*_*ϕ*_(*x*) and *F*_*ϕ*_(*y*) be true. By the second conjunct of the definition of a mereological fusion, *x* ≼ *y* and similarly *y* ≼ *x*. But then by antisymmetry *x* = *y*.

Define the partial function *f* such that for any *x* such that ∃*y x* ∼ *y*, *f*(*x*) is just that unique *z* such that *F*_*x*∼*y*_(*z*), that is, the mereological sum of all the objects ∼ comparable to *x*. Leave *f* undefined if there is no *y* such that *x* ∼ *y*. Because ∼ is a partial equivalence relation, it therefore will follow *f*(*x*) = *f*(*y*) ↔ *x* ∼ *y*.

Thus, for any partial equivalence relation ∼, one may define a new class of objects that are the mereological sums of the ∼ equivalence classes and a function that takes objects to their ∼ equivalence sums.

But one such example of a partial equivalence relation over established bodies of matter is the bodily contiguity relation that Linnebo outlines in *Thin Objects*. This can be used in the above manner to define ordinary bodies as the mereological sums of bodily contiguous blocks of matter. Consequently, reference to ordinary bodies can be explained mereologically and without relying on **RBA**.

The nominalist therefore has their alternative explanation for the tricky case of reference to ordinary objects. By accepting the axiom of fusion and antisymmetry, the nominalist may show that abstraction principles can secure reference but only between members of an equivalence class and their mereological fusion. But assuming that the mereological sums of non‐abstract objects are also non‐abstract, it follows that mereological abstraction is not strong enough to secure reference to abstract objects via abstraction from ordinary objects. The nominalist can explain Linnebo's ordinary bodies case as an instance of mereological abstraction using bodily contiguity and does so without committing to the existence of abstract objects.

#### Why accept the axiom of fusion?

Whilst there is a great deal of debate over the axiom of antisymmetry (see Varzi and Cotnoir (2021) section 4.2 for a survey), its role is really only to ensure unique mereological fusions. The axiom of fusion is clearly the more controversial premise here. The nominalist can defend the axiom of fusion in two ways. First, they can provide a defence of the axiom tout court. If such an argument were to be successful, it would leave Linnebo in a difficult position. If there is a compelling argument for the axiom of fusion and the axiom of antisymmetry that does not rely on **RBA**, then there is already explanation of reference to ordinary bodies and an explanation via **RBA** would be unnecessary. Quite simply, there would be no puzzling case left to explain, and Linnebo would lose the strongest part of his abductive argument. Second, just as Linnebo argues for **RBA** via abduction from cases like reference to ordinary bodies, so too can the nominalist argue for the axiom of fusion abductively from the ordinary bodies case. The burden will fall on them to show that their explanation via the axiom of fusion is at least as good as Linnebo's explanation via **RBA**. Both options are explored.

##### A defence of the axiom of fusion tout court

Unlike **RBA**, the axiom of fusion follows from the flexible conception of ontology.

As stated above, the flexible conception is understood here as the claim that any specifiable portion of reality is an object.

The axiom of fusion takes the form of a conditional based around some defining formulae *ϕ*, with appropriate free variables. The antecedent is the claim that there is at least something that does satisfy *ϕ*, and the consequent is the claim that there is some unique entity that is the mereological fusion of the *ϕ*s. There are two cases: when the antecedent is satisfied and when it is not. Clearly, then the antecedent is not satisfied, the conditional is uninterestingly true. The interesting case is where there is some formulae *ϕ* of the appropriate form and some *x* that satisfies *ϕ*, given some assignment to the other variables in *ϕ*. In this case, the flexible conception of ontology entails the existence of the mereological fusion of the *ϕ*s.

Let *ϕ* be a formula of the appropriate form. Because *ϕ* has *x* free, it is a description. Possibly, *ϕ* is a definite description, if it is satisfied by exactly one thing. Possibly it is an indefinite description if many things satisfy *ϕ*. Either way, *ϕ* has succeeded in specifying a portion of reality via description. The flexible conception entails, then, that this portion of reality is an object. But this object is clearly the mereological fusion of the *ϕ*s. It is the portion of reality overlapping all and only the things that satisfy *ϕ*.

In short, one arrives at the following argument:

P1: Any way of specifying a section of reality succeeds in referring to that section. (Flexible conception)
P2: Reference to some section of reality is sufficient for the existence of an object corresponding to that section. (Linnebo's first premise)
P3: For any formulae *ϕ*(*x*) with *n* + 1 free variables and any assignment to the first *n* variables, if there is an *x* that satisfies *ϕ*(*x*), then *ϕ*(*x*) specifies a section of reality, namely the mereological fusion of the *ϕ*'s.
C: The axiom of fusion is true.

P1 and P2 are assumed in this paper, given that Linnebo agrees with both of them and advocates for them in *Thin Objects*.

P3 can be defended as follows. A relatively uncontroversial claim is that true definite descriptions are capable of picking out particular portions of reality, namely the singular thing that satisfies the description. If ∃!*x ϕ*(*x*), then *ϕ* succeeds in specifying some single entity. What is a little more controversial is that indefinite descriptions (or just descriptions generally) are capable of the same. Some indefinite description *ϕ*(*x*) such that ∃*xϕ*(*x*) succeeds in picking out the *ϕ*s, in just the same way that a definite description picks out a singular object. It is worth noting that it remains to be seen if those things actually form an object or not. P1 says that they are, but that is up for debate. But P3's claim is very minimal. It is just the claim that descriptions generally pick out a portion of reality, as they do in the special case of definite descriptions.

##### An abductive justification of the axiom of fusion

The mereological explanation of the ordinary bodies case is offered as a rival explanation to Linnebo's **RBA** explanation. The nominalist, therefore, does not necessarily need to provide a prior defence of the mereological principles they employ in the explanation if their aim is merely to offer the mereological explanation as a rival explanation of the case at hand. Naturally, external arguments like the one above only help their case, but they are not strictly necessary.

The above section “reference by mereology” shows that the axiom of fusion and axiom of antisymmetry together do succeed in explaining the reference to ordinary bodies case. What remains to be argued is that this explanation is at least as good as Linnebo's.

The case in favour of the mereological explanation is rather intuitive, as is the case against it.

**RBA** has incredibly strong consequences in terms of the sorts and volume of objects it entails. By Linnebo's own demonstration in *Thin Objects*. **RBA** entails higher infinities of sets. Moreover, **RBA** involves a commitment to abstracta. Even if one is not a nominalist, one can still recognise avoiding unnecessary use of abstract objects as an abductive virtue. It is difficult to overstate just how strong the consequences of **RBA** are. The nominalist explanation explains Linnebo's most puzzling case without such heavy commitments.

There are two possible vices of the nominalist explanation.

The first mirrors Linnebo's own criticism of a particular nominalist strategy not endorsed by this paper. One of his nominalist strategies is to accept a local version of **RBA** in the case of ordinary bodies, but just deny it generally. Rightfully, Linnebo argues that this is ad hoc. A similar objection might be levelled against this paper's explanation.^8^ However, this would be unreasonable. Mereological principles have a wide range of explanatory power and seem to capture what goes on in a great many cases across a wide range of areas. Employing such a tool is therefore systematic and general as opposed to ad hoc.

Second, an advantage that **RBA** has over the nominalist explanation is that it explains what happens in a wide range of cases. Aside from the kinds of cases discussed here, **RBA** also provides a nice explanation as to why mathematical statements are true: it entails the existence of a range of abstract objects that model those statements. As is mentioned above, the nominalist has a more disunified approach than Linnebo's. The nominalist has to explain each of these cases separately and often employs different mechanisms in each one. What Linnebo's explanation offers is a kind of theoretical unity that the nominalist cannot match. This is a genuine and interesting theoretical virtue that Linnebo's position offers that the nominalist view does not.

However, how one ought to weigh theoretical unity against parsimony is a complex question with no clear answer in sight. This is an ongoing and open philosophical question. It is for this reason that this paper stops short of claiming that the nominalist explanation of the cases at hand is clearly better than Linnebo's. There is clearly something to be said in favour of Linnebo's explanation via **RBA**. But **RBA** comes with serious costs. It is an exceptionally unparsimonious explanation of a relatively mundane event. Avoiding that commitment is a benefit that ought not be taken lightly.

Thus, both views seem to have a comparably strong consideration counting in their favour. This is a good reason to think that the nominalist explanation is at least as good as Linnebo's, and hence the nominalist is permitted to accept this explanation of the ordinary bodies case and avoid commitment to **RBA** and hence abstracta.

## 4 CONCLUSION

This paper provides a nominalist response to Øystein Linnebo's argument for the existence of abstract objects via abstraction. The crucial premise that the response targets is the second premise, **RBA**. Linnebo's argument for this premise is taken to have two parts (Section 2): a deductive and an abductive. The deductive part is argued to be unsound (Section 2), and a rival nominalist explanation is presented (Section 3) for the abductive argument. The paper only argues for a weaker amicable conclusion that the nominalist is justified in rejecting **RBA**, irrespective of whether Linnebo is justified in accepting it.

A stronger claim would be that this creates a problem for Linnebo, not just a way out for the nominalist. It might be the case that (1) Linnebo's commitment to the flexible conception of ontology entails the mereological principles that explain the ordinary bodies case or (2) that on closer analysis the nominalist explanation is clearly better than Linnebo's, thereby blocking his abductive inference. This stronger claim, however, is left to future work. For this paper, suffice to say that the nominalist has a way out of Linnebo's argument by rejecting **RBA**.

## Footnotes

^1^
Abstraction is not new. See for instance Frege (1884), Wright and Hale (2001), or Fine (2002). Something akin to dynamic abstraction is present in Fine's work. Linnebo's contribution here is the thoroughness and novelty of this particular argument for dynamic abstraction, the working out of its consequences and the application of the notion of thinness.

^2^
This paper avoids a discussion as to what the correct definition of “abstract” actually is. Suffice to say that any worthwhile definition of “abstract” would include sets and Linnebo's argument from dynamic abstraction entails the existence of sets. See Burgess and Rosen (1997) for discussion on different definitions of “abstract.”

^3^
This paper does not target Linnebo's claims about the thinness of these objects. The conclusions that (1) these objects exist and (2) these objects are thin, are separate, and need to be taken separately. If the nominalist is wrong and these objects do exist, I find Linnebo's argument that they are thin highly persuasive. My thanks to a reviewer for asking for clarity on this point.

^4^
The epistemic nominalist, however, needs to make the stronger claim that Linnebo's abductive inference is invalidated by the presence of the nominalist explanation. This is either because (1) the nominalist explanation is better or (2) conservative abduction principles apply. Personally, I am convinced by (1) and not (2), though will not argue for this conclusion here.

^5^
Typically, these objects are abstract, e.g. sets. However, on Linnebo's view, abstraction does not always lead to abstracta. His case of reference to ordinary bodies is one such example. See §2.2 for discussion.

^6^
Let *sim*_*T*_ be a non‐transitive similarity relation such a biological similarity. Let A be a class whose members have *T* similar relatives and represent archetypal members of each desired *T*‐type. Define a transitive similarity relation ${\mathrm{sim}}_{\left\langle T,A\right\rangle }$ such that ${\mathrm{sim}}_{\left\langle T,A\right\rangle }\left(x,y\right)$ is true iff the member of **A** that *x* and *y* are most similar to is the same. Ties are broken arbitrarily.

^7^
A weaker version of the axiom of fusion requires *ϕ* to have only *x* free and dispenses with the universal quantifiers over the other variables. Varzi and Cotnoir (2021) do not discuss the difference between these two versions. In models where all objects are designated by some term, the two formulations collapse into one another. The stronger formulation is necessary for this paper because *x* ∼ *y* has two free variables.

^8^
My thanks to a reviewer for asking for clarity on this point.
